# Current trends and future directions of global research on wastewater to energy: a bibliometric analysis and review

**DOI:** 10.1007/s11356-024-32560-2

**Published:** 2024-02-24

**Authors:** Zhining Shi, Ke Xing, Rameez Rameezdeen, Christopher W. K. Chow

**Affiliations:** UniSA STEM, University of South Australia, Mawson Lakes, SA 5095 Australia

**Keywords:** Wastewater, Energy, Anaerobic, Biogas, Hydrogen, Bibliometric analysis

## Abstract

This paper presents a structured bibliometric analysis and review of the research publications recorded in the Web of Science database from 2000 to 2023 to methodically examine the landscape and development of the ‘wastewater to energy’ research field in relation to global trends, potential hotspots, and future research directions. The study highlights three main research themes in ‘wastewater to energy’, which are biogas production through anaerobic digestion of sewage sludge, methane generation from microbial wastewater treatment, and hydrogen production from biomass. The analysis reveals activated sludge, biochar, biomethane, biogas upgrading, hydrogen, and circular economy as key topics increasingly gaining momentum in recent research publications as well as representing potential future research directions. The findings also signify transformation to SDGs and circular economy practices, through the integration of on-site renewables and biogas upgrading for energy self-sufficiency, optimising energy recovery from wastewater treatment systems, and fostering research and innovation in ‘wastewater to energy’ supported by policy incentives. By shedding light on emerging trends, cross-cutting themes, and potential policy implications, this study contributes to informing both knowledge and practices of the ‘wastewater to energy’ research community.

## Introduction 

Wastewater management consists of a series of energy-intensity processes, including collection, conveyance, treatment processes, and sludge disposal. Wastewater treatment alone consumes about 25% of the electricity consumption of the water sector globally and contributes to 3% of greenhouse gas emissions (IEA 2018). Energy efficiency and energy savings are essential to the wastewater sector to reduce operational costs and environmental impact (Zuo et al. 2023). A wide range of studies has focused on techniques to increase energy efficiency and lower the operational costs of wastewater treatment plants (WWTPs). Some water utilities, such as SA Water in South Australia, even set ambitious targets to achieve zero energy costs (AWA 2019). Wastewater contains five to ten times the amount of energy required for the treatment process and is a potential source of biohydrogen (WRF 2021). In recent years, wastewater has been increasingly studied as a source of clean energy for industrial WWTPs, which contributes to energy optimisation of wastewater treatment and the reduction of carbon emissions.

Wastewater can be used to generate biogas, biomethane and green hydrogen with technologies include biomass gasification (Cao et al. 2020), photo-fermentation (Cheng et al. 2011), photocatalysis (Rasouli et al. 2023), electrolysis (Zeppilli et al. 2019), biosolid pyrolysis (Patel et al. 2020), and supercritical water gasification (Shan et al. 2021). Some water utilities have been recovering energy as biogas through wastewater treatment processes to generate electricity, which covers up to 85% of the energy demand for WWTPs (WSAA 2012). Innovative technologies have been developed to turn biogas into hydrogen, such as the use of iron ore (ARENA 2019) or biochar (Patel et al. 2020) as catalysts. There have been reviews conducted on energy recovery from wastewater, most of which focus on specific topics, including biogas production from anaerobic digestion of sewage sludge for on-demand electricity generation (Lafratta et al. 2020), methane production from sewage sludge (Choi et al. 2018), aerobic granular sludge (Purba et al. 2022), wastewater treatment for sustainable water resources management (Silva 2023), and microbial electrochemical technologies (Deng et al. 2023). Bio-hydrogen production from wastewater (Islam et al. 2021) and resource recovery from municipal WWTPs as water, energy, nitrogen, and phosphorus (Kehrein et al. 2020) have also been examined.

While the number of publications on ‘wastewater to energy’ research has been growing significantly in recent years, those studies were predominantly focused on technology development, applications, and evaluation for wastewater treatment and energy conversion. In contrast, there is a lack of methodical analysis on the development of the ‘wastewater to energy’ research field to identify global trends, potential hotspots, and future research directions in this aspect. To address such limitations, bibliometric analysis represents the befitting technique for quantitative and qualitative analysis of patterns and characteristics of scientific publications (Zhang et al. 2017). It has been widely used in recent years to visualise qualitative and quantitative information (Goh and See 2021) as well as evaluate research trends based on published articles (Saidulu et al. 2021). The technique often serves to examine topics, themes, and research methods in a particular field and the trends of the research, including the wastewater area (Mao et al. 2021). For example, in the field of wastewater treatment, Mao et al. (2021) applied bibliometric analysis, combined with other techniques such as social network analysis, to study global trends in industrial wastewater treatment and treatment technologies from 1998 to 2019. Similarly, the bibliometric method was also used to benchmark the scientific research on wastewater-energy nexus for energy and resources recovery prior to 2015 (Zheng et al. 2017). In addition, Marcal et al. (2021) adopted this method to examine European municipal wastewater research in the 2010s and identified a paradigm shift from pollutant removal to resource recovery. Although the aforementioned studies performed bibliometric analysis on ‘wastewater to energy’ research publications over time, there are still limitations and gaps in this area. Despite having the wastewater and energy nexus covered in the analysis, it was only examined as part of the scope of reviewing research on wastewater treatment processes and technologies, rather than a dedicated area of focus. Therefore, the analysis of research patterns and trends in this particular aspect was rather limited and less comprehensive. For those bibliographic analyses centred specifically on ‘wastewater to energy’, the studies reported so far in extant literature were largely based on research work and outcomes up to the first half of the 2010s and mostly limited to technology development for biogas and methane production from wastewater treatment processes. There is still a lack of clear understanding and systematic analysis of the overall research landscape and progression trends in the ‘wastewater to energy’ field including biogas, methane, and hydrogen generation from biomass, biosolids, or sludge of wastewater. Hence, a new study is much needed and especially useful to inform knowledge and practice by comparing with earlier analyses reported to shed light on hot issues, emerging trends, cross-cutting themes, and potential policy implications for the ‘wastewater to energy’ research community. With these in mind, this paper is set out to explore and address the following four questions about the ‘wastewater to energy’ research:

- What have been the overall global trends for the past two decades?
- How have the research focuses been evolving over time?
- What has the recent progress in research been?
- What are the likely trends and hotspots for future research?

## Methods

A bibliometric analysis approach was adopted in this study to provide a methodical investigation of research literature on energy generation, particularly in the forms of biogas and hydrogen, from wastewater over the past two decades. The information used to interrogate publication patterns in the literature included keywords, categories of articles, journals, and countries of publications. Word-cloud analysis, keyword occurrence and co-occurrence analysis, and keyword network analysis were performed to help explore global research trends and identify hotspots of ‘wastewater to energy’ research.

### Data collection

**Fig. 1:**
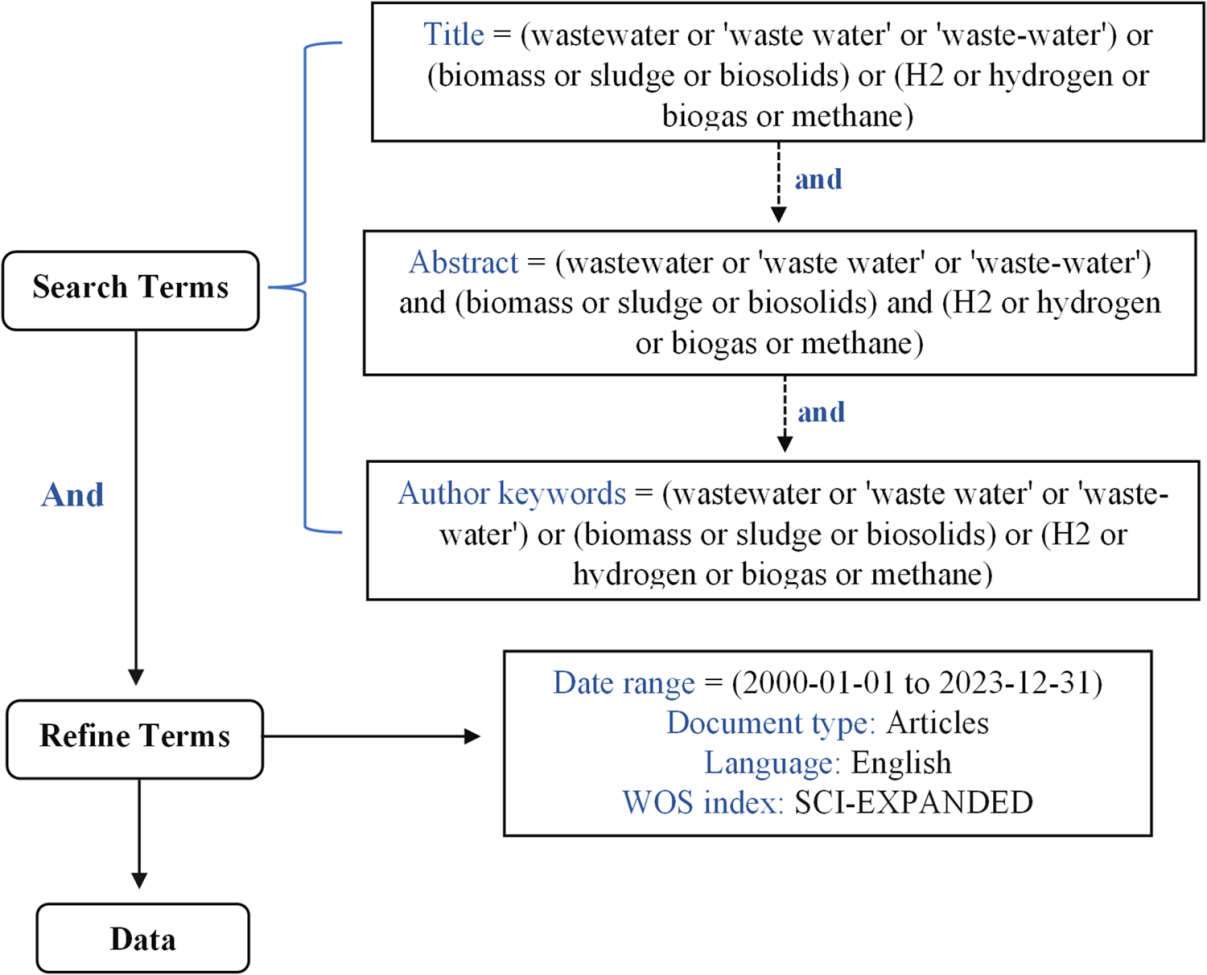
The search strategy for 'wastewater to energy' research literature

Bibliometric analysis is a widely used research method to analyse the state of the art for a particular field in publications. The bibliographic data for this study were retrieved from the Web of Science (WoS) Core Collection database. The WoS is one of the most comprehensive databases used by past studies of bibliometric analyses on wastewater, which includes a comparative study of low energy–intensive production processes for hydrogen generation from wastewater (Islam et al. 2021) and an analysis of industrial wastewater treatment trends (Mao et al. 2021). The data utilised in this study was first extracted using search queries on 18 January 2023 and then updated on 18 January 2024. The search queries were performed using the ‘front page’ filter technique which the queries search in titles, abstracts, and author keywords of articles to screen the relevant documents effectively (Ho 2020). This technique has been used to analyse emerging interest in water research and research collaborations (Goh and See 2021). The search queries are presented in Fig. 1 and limited to English language and Science Citation Index Expanded articles. The ‘wastewater to energy’ in this study was defined as ‘biomass or sludge or biosolids’ of wastewater converted to energy as ‘hydrogen or biogas or methane’. Thus, the keywords for collecting publications were defined as (wastewater or ‘waste water’ or ‘waste-water’) and (biomass or sludge or biosolids) and (H_2_ or hydrogen or biogas or methane). The timespan of publications was set from 1st of January 2000 to 31st of December 2023. Data of these publications during this period were extracted and pre-treated to remove the duplicates or irrelevant information before being admitted for further analysis.

### Data analysis and in-depth review

**Fig. 2:**
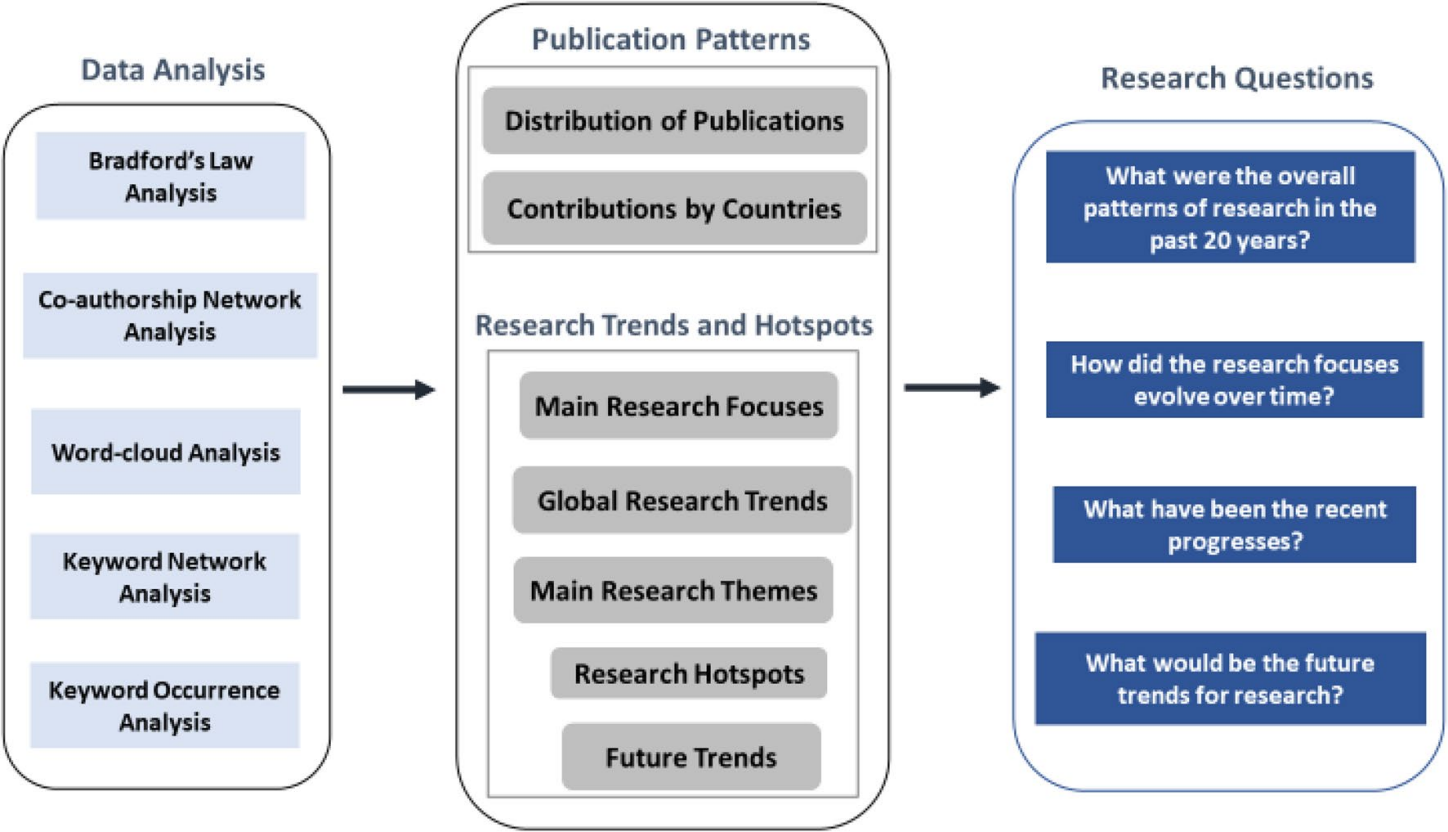
Data analysis methods used to address the research questions

A range of data analysis methods was applied as shown in Fig. 2 to answer the research questions of this study. Both quantitative and qualitative bibliometric analyses were performed to interrogate the collected data. Firstly, the overall publication patterns were analysed based on the year of publication, journals, and the country of the research conducted, to cast light on the distribution and concentrations of the research work. Then, research themes and trends were further analysed by examining a word-cloud of keywords, probing the co-authorship networks, mapping knowledge structure by co-occurrence with author keywords, and inquiring into the most cited publications.

The information used for overviews of the publication patterns in the area of ‘wastewater to energy’ was mainly extracted from the collected publication data using the Biblioshiny’ and the Bibliometrix package. The Biblioshiny provides a web interface for the Bibliometrix (Aria and Cuccurullo 2017) under R version 3.6.2. The package is an open-source tool for quantitative research that has been used for various studies to extract useful information for research trends analysis since 2017. The overview of the publications includes the total number of publications, annual scientific production, the annual increase of the publications, and the number of articles for the top WoS categories per year in the field of ‘wastewater to energy’.

The information on the top ten core sources of ‘wastewater to energy’ research was identified by using Bradford’s Law. Bradford’s law describes how publications are distributed in journals, which has been widely employed in bibliometric research to identify the most-cited journal in a field (Marcal et al. 2021). Publication contributions by countries were analysed based on the countries of corresponding authors to determine the countries with the most journal articles. A map of scientific advances in the ‘wastewater to energy’ field was generated for the visualisation of publication patterns based on the countries’ publications using the Datawrapper, which is an open-source web tool for creating interactive graphs.

A word-cloud is generated using the Biblioshiny to analyse prominent keywords in the field of wastewater to energy. The size of the word in the word-cloud depends on the occurrence frequencies of the keywords shown in the articles. The word-cloud allows quick visualisation by highlighting the most frequently used keywords in all the publications.

The co-authorship network for the top 20 countries was analysed in terms of the number of publications with the full counting method. The full counting method is that a publication that has co-authors from multiple countries is counted as a full publication for each of those countries. A co-occurrence network with author keywords was also generated based on cluster analysis to show the number of publications that appear together using the same keywords. Cluster analysis establishes similarities between keywords and has been widely used to explore the research trends from published articles (Zheng et al. 2015). The co-authorship and co-occurrence networks were generated by employing VOSViewer (version 1.6.17), which is a computer-aided tool to construct networks of scientific publications (Tan et al. 2021) and has been used in research to visualise bibliometric networks (Marcal et al. 2021).

## Analysis of publication patterns

By following the process depicted in Fig. 1, the dataset derived from the literature survey in the field of ‘wastewater to energy’ contains 2539 published journal articles in 303 sources with 7328 authors and 5861 keywords, indicating this is a highly active research field. In terms of authorship, the average number of authors per article is 3. There are 7276 authors of multi-authored articles, with only 60 single-authored articles, showing a strong sign of collaborative work in this research field.

### Overview of publications

**Fig. 3:**
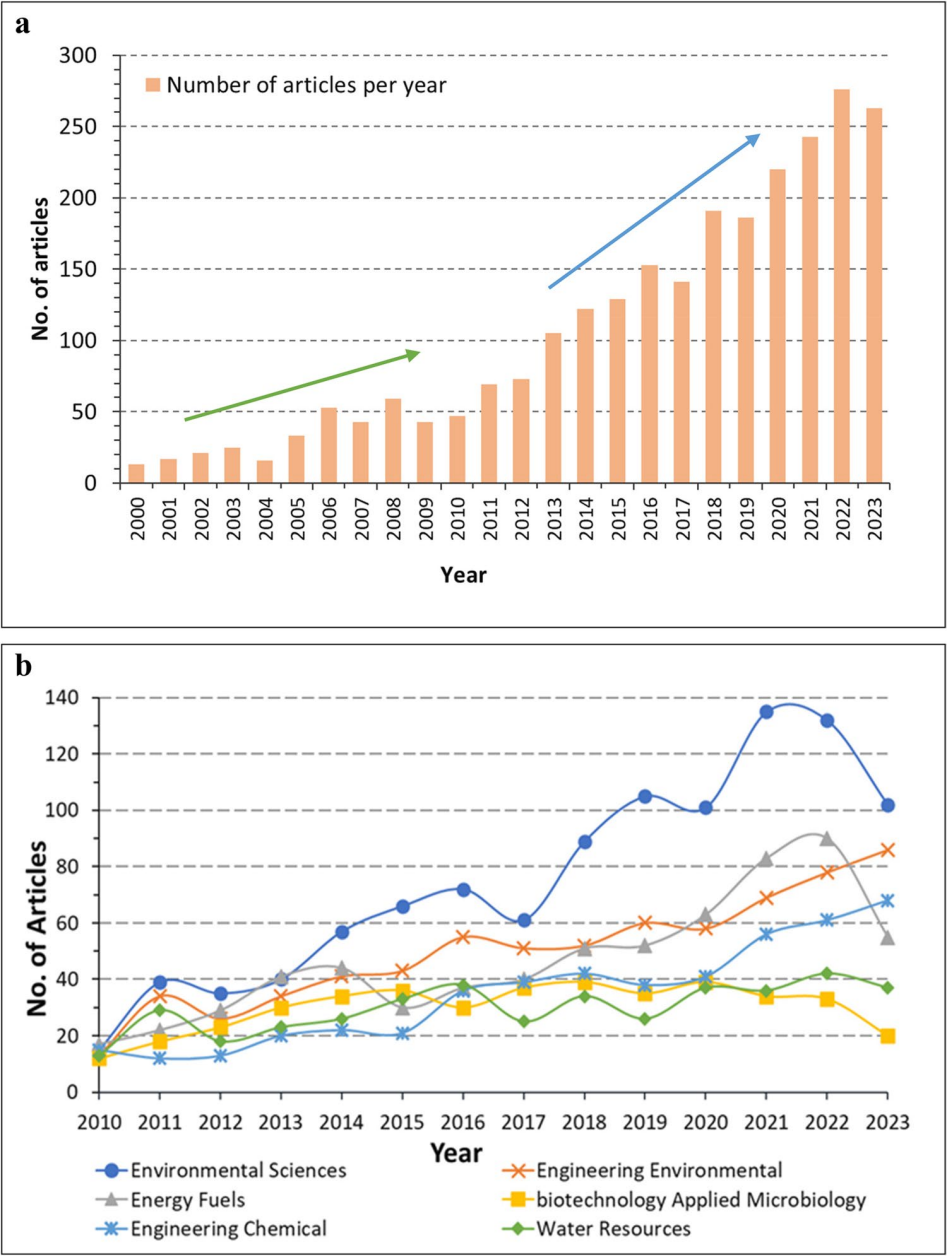
**a** Annual scientific production per year from 2000 to 2023 and **b** number of publications for top WoS categories per year in the field of ‘wastewater to energy’ from 2010 to 2023

As depicted by the number of articles published per year during 2000–2023 (Fig. 3a), the total number of publications related to the wastewater and energy nexus has grown from only 14 in 2000 to more than 260 in 2022 and in 2023, with an average annual growth rate of 17.5%. This demonstrates a clear trend of dramatic progress and increase in publications from 2010 and onward in contrast to the small scale of research in the first 10 years of this millennium. The six main WoS categories of studies on ‘wastewater to energy’ and the number of publications in each of the respective categories between 2010 and 2023 are presented in Fig. 3b to reveal the trends of research progress in the relevant disciplines over the recent decade. *Environmental Sciences* appears as the top category for the area of analysis, with the number of published articles in the category significantly escalating since 2013 and a much higher publication rate compared to the other categories. A steady increase was observed in the number of publications in *Engineering Environmental* and *Energy Fuels* over the past decade*.* In contrast, rather fluctuating trends of publications were noticed in the categories of *Biotechnology Applied Microbiology*, *Engineering Chemical*, and *Water Resources*. Such trends suggest that the progress of research developments in ‘wastewater to energy’ shows signs of diversification in interests, from traditional focuses on wastewater treatments to more cross-cutting themes of sustainability and clean energy research.

Noticing that over 70% of the articles published were between 2015 and 2023, this serves as a clear manifestation of growing and strong interest in ‘wastewater to energy’ topics and studies in recent years. The dramatic increase in this field of research can be attributed to countries’ emissions reduction targets, e.g. the European Union’s greenhouse gas emissions target of 20% reduction by 2020 (Scarlat et al. 2015) and China’s progressive policies and plans for water pollution control since 2014 (Xu et al. 2020). An increasing number of water utilities are now moving towards ‘net zero’ strategies to minimise carbon emissions and tackle climate change by exploiting more clean and renewable energy sources and optimising energy consumption (Ballard et al. 2018).

The top ten core sources of ‘wastewater to energy’ research were identified using Bradford’s law and are shown in Table 1, sorted according to the cumulated number of publications. Overall, these ten sources cover 43.1% of the total number of relevant articles through 2000–2023. Among those journals, *Bioresource Technology* and *Water Science and Technology* emerged as the two leading sources for ‘wastewater to energy’ research publications.

**Table 1:** The top ten core sources of 'wastewater to energy' research publications in 2000–2023

Core sources	Cumulated number of publications (%)	Impact factor	Global h*-*index	*WOS category*
Bioresource Technology	211 (8.3)	11.4	341	Agriculture engineering; Biotechnology applied microbiology; Energy fuels
Water Science and Technology	164 (6.5)	2.7	153	Engineering environmental; environmental science; Water resources
Water Research	148 (5.8)	12.8	354	Engineering environmental; environmental science; Water resources
International Journal of Hydrogen Energy	135 (5.3)	7.2	248	Chemistry physical; Electrochemistry; Energy fuels
Journal of Environmental Management	100 (3.9)	8.7	218	Environmental sciences
Science of the Total Environment	84 (3.3)	9.8	317	Environmental Chemistry; Environmental Engineering
Chemical Engineering Journal	80 (3.1)	15.1	280	Engineering chemical; Engineering environmental
Journal of Cleaner Production	68 (2.7)	11.1	268	Engineering environmental, environmental science; Green sustainable science technology
Renewable Energy	53 (2.1)	8.7	232	Energy fuels; Green sustainable science technology
Environmental Technology	52 (2.0)	2.8	84	Environmental sciences

Note: Impact factors of the journals were extracted from the Journal Citation Reports, and the h-index was retrieved from Scimago Journal & Country Rankings on the 18th of January 2024

### Contributions by countries

**Fig. 4:**
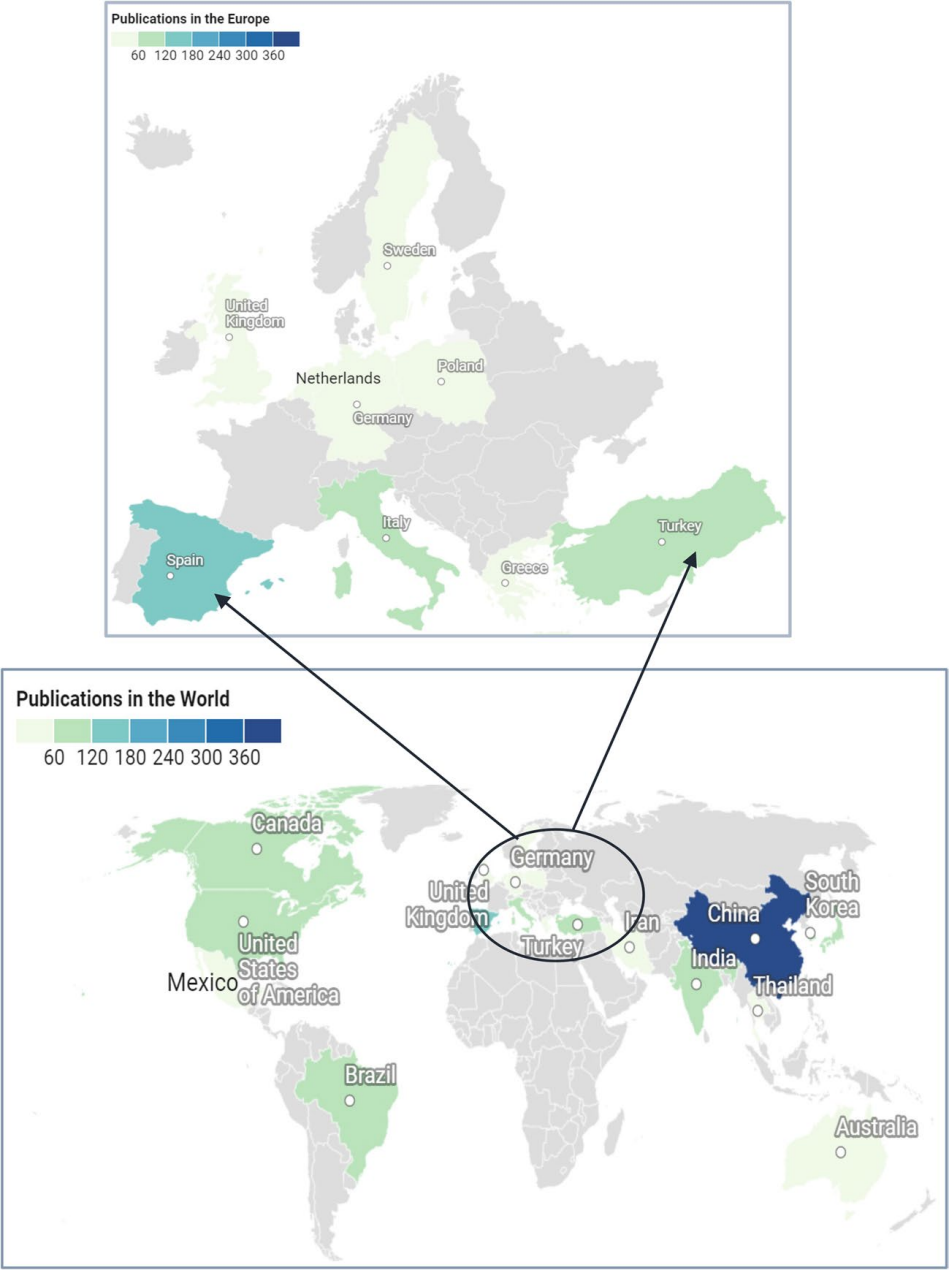
Thematic maps of scientific advances on ‘wastewater to energy’ of the top 20 countries

Based on the nations of the corresponding authors, the top 20 countries with the highest research output during the study period and their geographical distributions are shown in Fig. 4. It is discerned that China had 602 articles contributing to ‘wastewater to energy’, equivalent to more than 20% of the total publications in this research field globally. This represented the highest concentration of research, followed by Spain (153), India (140), the USA (127), and Japan (119). The highest research output from China could be attributed to intensive development in wastewater treatment due to progressive government policies in water management and pollution control since 2014 (Neighbour 2020) and continuously growing financial support (Qu et al. 2019). This is consistent with China’s strong innovation for the future revolution of wastewater management and treatment technologies and towards new wastewater treatments focusing on energy recovery from sludge (Xu et al. 2020) and carbon reduction since 2014 to meet the target of carbon neutrality by 2030 (CEWP 2021).

**Fig. 5:**
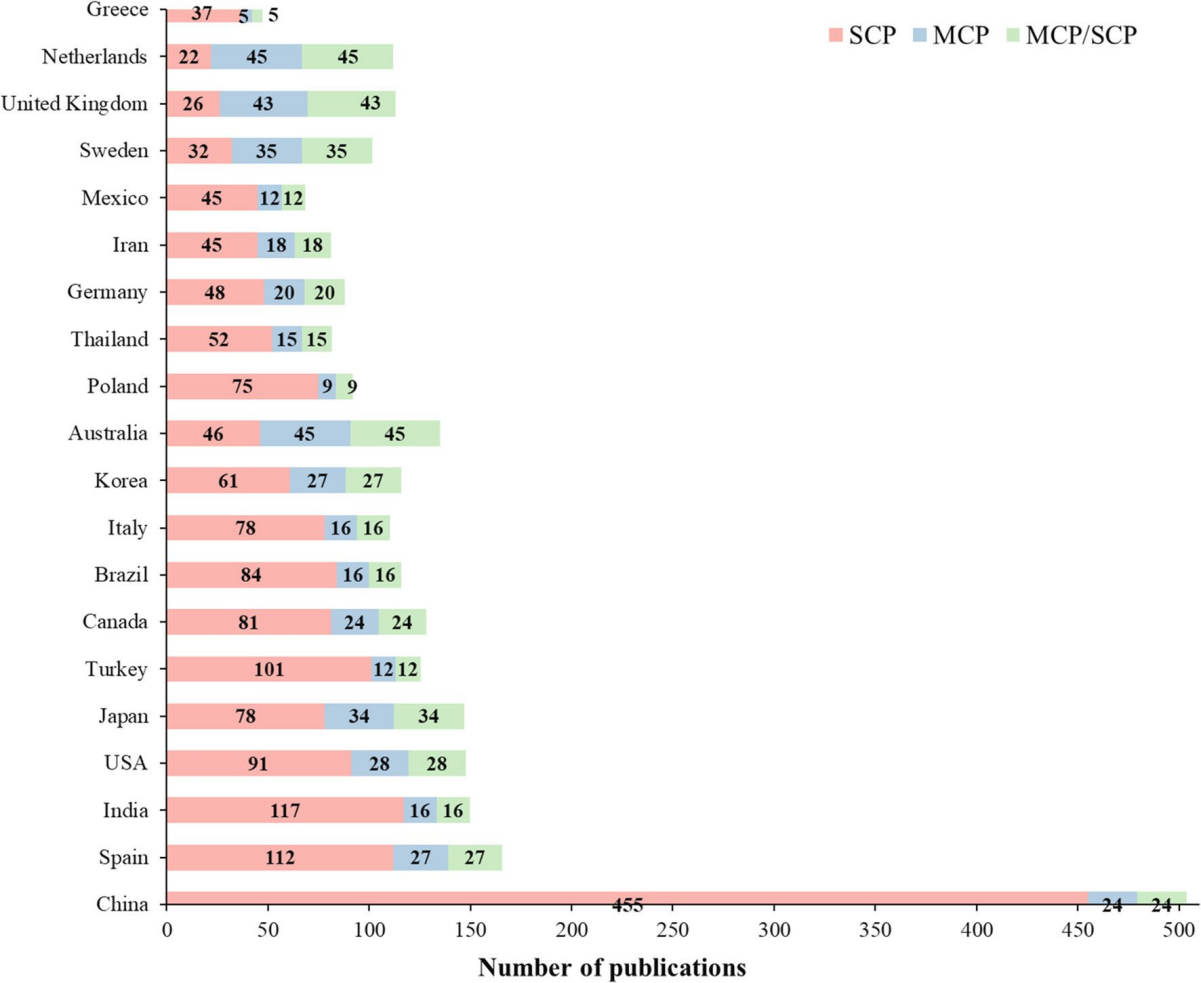
Single-country publications (SCP), multiple country publications (MCP), and the percentage of MCP over the total number of publications of the corresponding author’s country for the top 20 countries with the highest research outputs for ‘wastewater to energy’

By further analysing the authorship, publications were further categorised into single country publications (SCP), i.e. collaborations of authors within the same country, and multiple country publications (MCP), i.e. collaborations of authors across two or more countries, as shown in Fig. 5. China was found to have the highest SCP and MCP among all the countries. The MCP/SCP ratio in Australia and the Netherlands was the highest among the countries studied, reaching 45%, indicating that nearly half of the publications from these countries involved international collaboration. Followed by the UK, Sweden, and Japan, which had MCP and SCP ratios of 43%, 35%, and 34%, respectively. These countries with high ratios of MCP and SCP demonstrated great interest in international collaborations in the ‘wastewater to energy’ area.

**Fig. 6:**
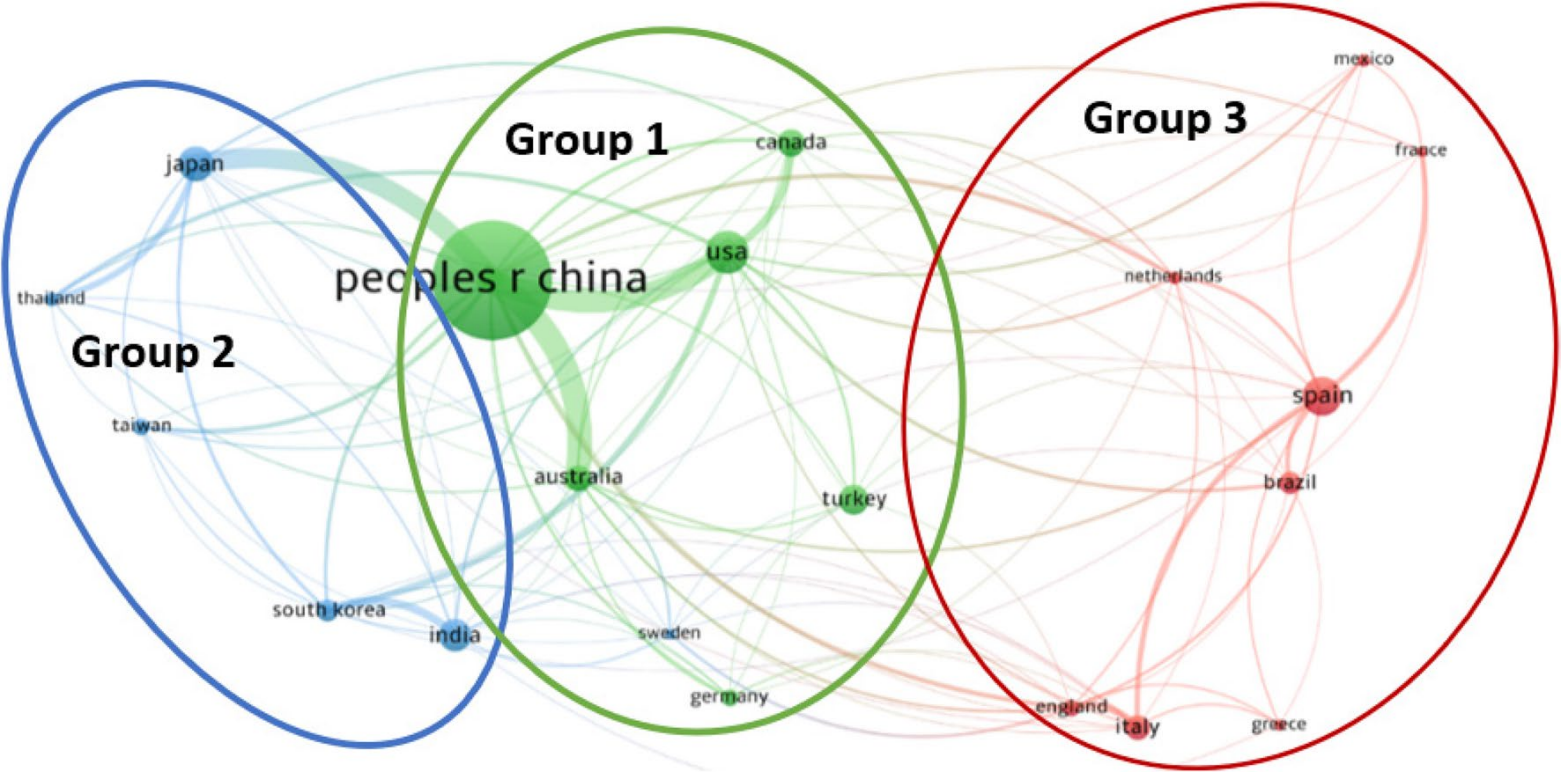
Co-authorship network for the top 20 countries in terms of the number of publications. Note: ‘people r china’ refers to ‘People’s Republic of China’, which is the official name of China

A co-authorship network, as demonstrated in Fig. 6, was generated to examine the international academic collaborations among the top 20 countries. The full counting method was applied for the co-authorship network analysis with a threshold value of 30 applied as the cutoff for the minimum number of collaborative publications attributed to a country. The size of a node, which corresponds to a country, represents the number of publications produced by the particular country. Signifying the collaboration, the thickness of a link between two nodes denotes the strength of joint research, demonstrated by the number of co-authored publications, between the two countries. For ease of analysis and comparison, the 20 countries (nodes) were categorised into three groups, based on the extent of collaborations. Indicated in different colours, the formation of the network centred on China, Spain, India, and the USA, which demonstrated preeminent roles in contributing to the research collaborations and output within their respective subnetworks. It was also noticed that over the past 20 years, China, as the spotlight of Group 1, had extensive collaborations with the USA, Australia, and Japan. Japan and India were the key nodes of Group 2 and had more collaborations with South Korea, Thailand, and Taiwan. The USA had strong research links with China, Canada, and Turkey. As the key contributors of Group 3, Spain worked closely with Brazil, Italy, and the UK. Some European countries, such as Spain, Italy, the UK, Netherlands, France, and Switzerland, preferred cooperation within the European Union, forming a Europeanization of shared co-authorship (Mattsson et al. 2008). Consistent with the previous research on municipal wastewater research (Marcal et al. 2021), China can also be seen as the most collaborative country in the ‘wastewater to energy’ research field. The broad collaborations among the top 20 countries with the highest publications could be led by the tremendous growth in wastewater treatment research and substantial development of policies and plans in the area of pollution control since 2014 and energy recovery from sludge (Xu et al. 2020).

### Main research focuses

**Fig. 7:**
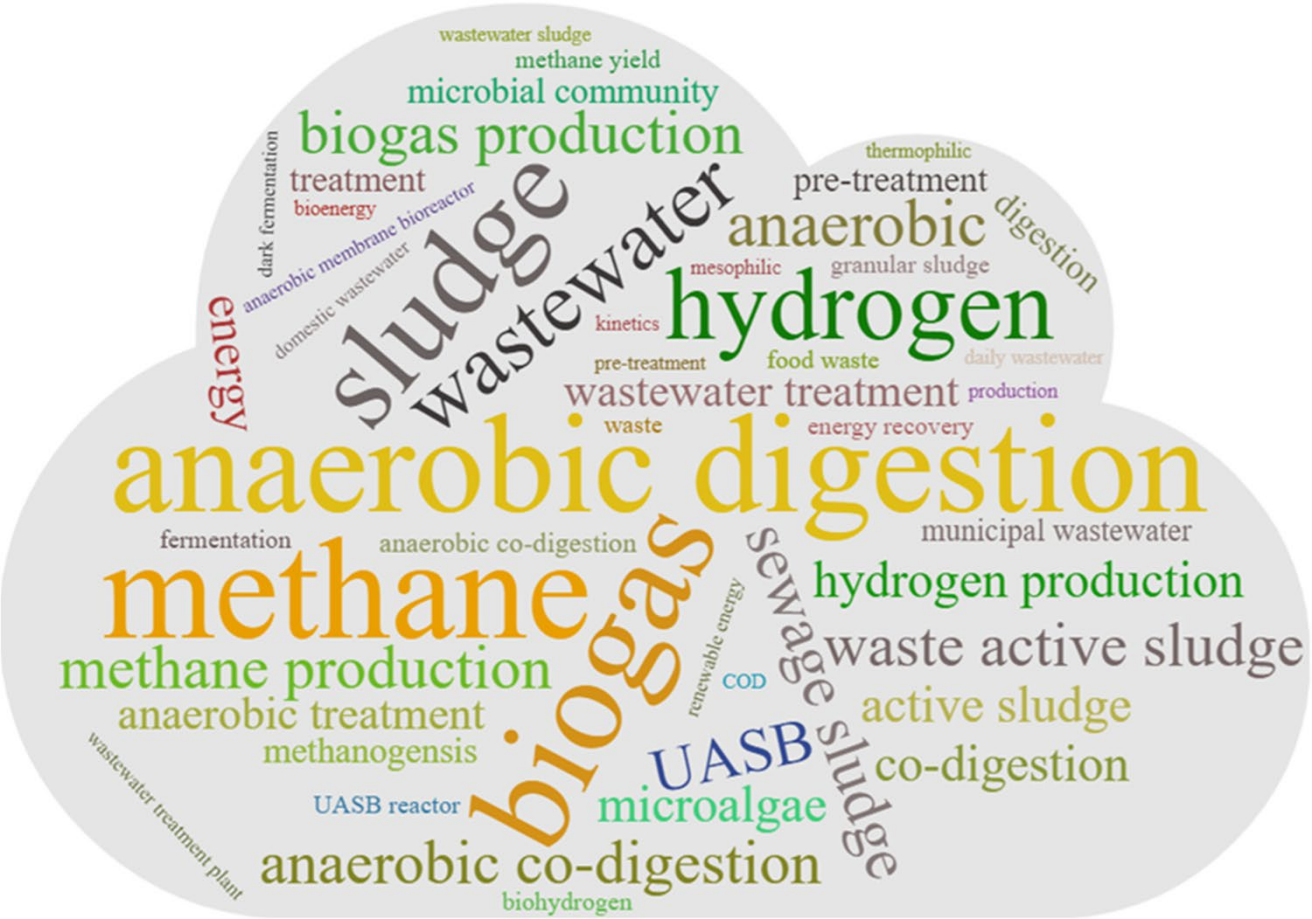
WordCloud of author’s keywords (top 50) based on the frequencies of keyword occurrence (2000–2023)

To enable identifying focal areas of research on the wastewater-energy nexus, a word-cloud diagram (Fig. 7) of author keywords was created to capture the 50 most frequently used keywords in all relevant publications over the past two decades. For a keyword, the higher the frequency of occurrence in publications, the larger the font size becomes to illustrate in the word-cloud. As shown in the diagram, *anaerobic digestion*, *biogas*, *wastewater*, *methane*, *sludge*, *sewage sludge*, *hydrogen*, and *wastewater treatment* represent the top keywords, each of which had the occurrence more than 100 times, among all the publications. *Anaerobic digestion* and *biogas* emerged as the top two keywords with the occurrence of 489 and 411 times, respectively, while *methane* and *hydrogen* also appeared 255 and 207 times each. The results of word-cloud analysis indicate that research on the ‘wastewater to energy’ had a strong focus on biogas generated from the anaerobic digestion processes and systems. Anaerobic digestion is a cost-effective and environmentally friendly technology for converting the organics of wastewater to biogas (Hanum et al. 2019). Anaerobic digestion attracted much focus of intensive innovation before 2017 (Zheng et al. 2017). Sludge is a source of green energy and some WWTPs have systems to recycle sludge into biogas for electricity in the European Union countries (Bodík et al. 2011), Brazil (Cañote et al. 2021), Poland (Masłoń, 2019), and Australia. Biogas includes methane (48–65%), and hydrogen can be used to generate energy for powering the WWTPs, which is important for resource recovery, energy savings, and carbon emission reduction.

## Analysis of trends and themes

### Global research trends

**Fig. 8:**
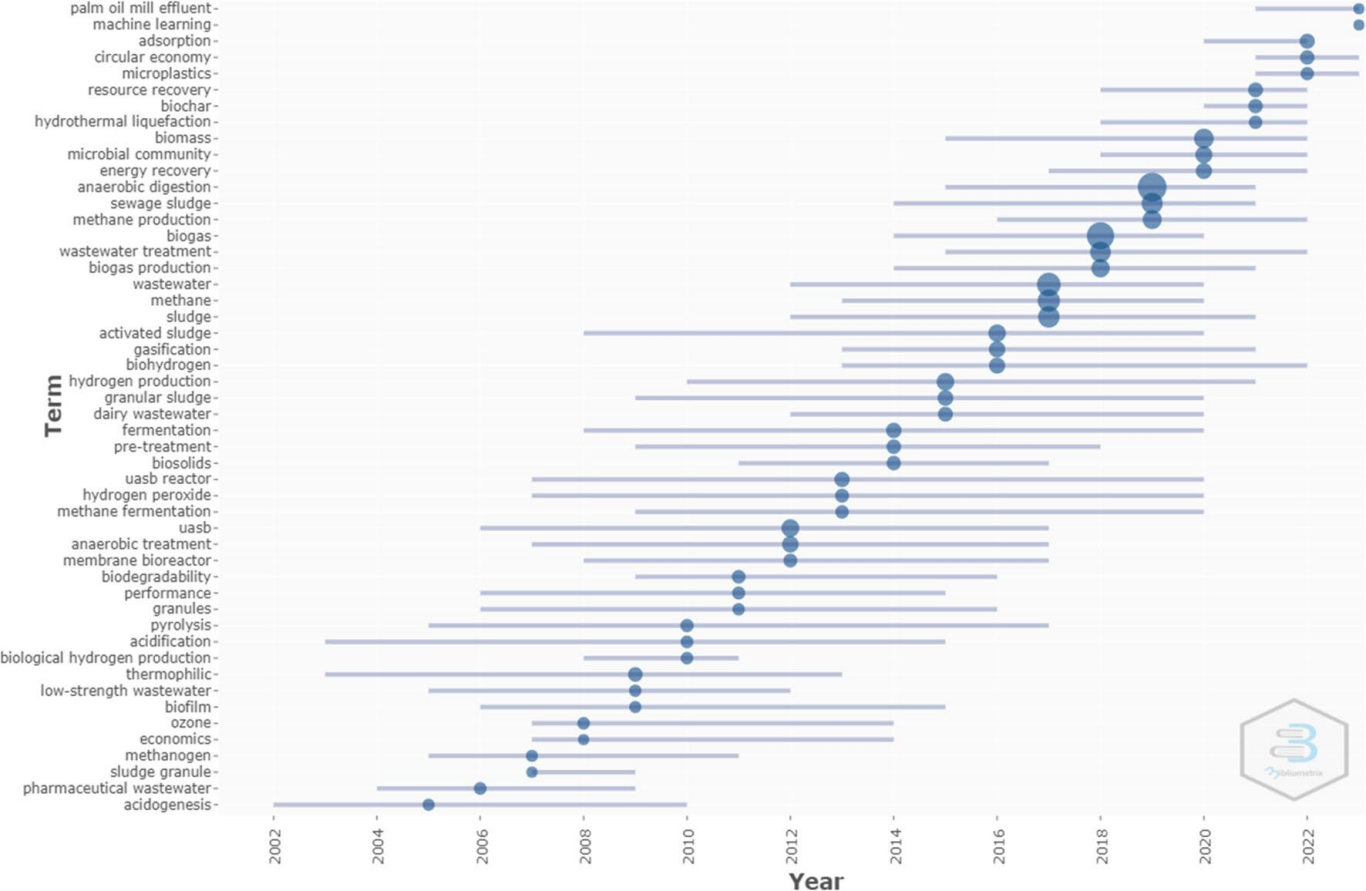
Research focuses on 'wastewater to energy' in the past two decades

Analysis of the occurrence frequencies of author keywords in the collected articles can help to further reveal how the ‘wastewater to energy’ research focus has evolved over time and the recent progress. The median year was used to select the keywords for each year. Figure 8 shows the evolution of research trends for the periods of 2000–2023 based on up to three keywords per year, with the condition of minimum frequency of occurrence set as five times.

The trend of ‘wastewater to energy’ research emerged in 2005, signified by *acidogenesis* (as one of the stages of anaerobic digestion and produces biogas from wastewater). While the frequencies of research were low (< 10) between 2005 and 2008, the research focus moved to *acidification* and *methanogen* of *pharmaceutical wastewater* in 2006. In 2007, the ‘wastewater to energy’ research appeared concentrating on *methanogen*, *clostridium*, and *flocculation*. The most popular research areas on wastewater to energy were *ozone*, *acidification*, and *hydraulic* in 2008. The trendy research topics in 2009 turned out to be *pH*, *thermophilic*, and *pyrolysis*. There was more research focused on *thermophilic* (appearance frequency of 26) than the other topics. Thermophilic often refers to a thermophilic wastewater treatment temperature of 50–60 °C and thermophilic bacteria grows at high temperatures. From 2010 to 2015, it occurred that research on wastewater to produce energy largely engaged in *anaerobic treatment*. *Biological hydrogen production* became a trendy focus for the first time in 2010, using the wastewater treatment technologies of *expanded granular sludge bed* with *granules*. The research in 2011 focused on *biodegradability* and performance of anaerobic wastewater treatment. Continuing with the trend, the research in 2012 and 2013 strong emphasis on the treatment technology of *upflow anaerobic sludge blanket reactor (UASB)*. Furthermore, *biosolids*, *pre-treatment*, and *fermentation* came to the research focus in 2014. Along with the high frequencies of *hydrogen production*, *granular sludge* and *daily wastewater* appeared in 2015, indicating rising intensive interest in research of hydrogen fuel production from wastewater anaerobic processes and gasification.

*Anaerobic digestion* and *biogas* became the most contemporary topics in ‘wastewater to energy’ research over the past decade. Between 2016 and 2019, keywords related to *anaerobic*, *biogas*, and *methane* appeared regularly as the largest number of occurrences in publications. This shows that biogas and methane produced from anaerobic digestion/co-digestion processes were significant research themes, fostered by substantial development of the anaerobic digestion technologies since 2010. The high occurrences of *wastewater*, *methane*, and *hydrogen* were considered to be related to energy recovery (Zhang and Li 2019) and bioenergy generation (Zhang and Li 2019). The concentrated research of wastewater to energy on biogas production of anaerobic digestion could be attributed to progressive policies and plans for wastewater treatment and renewable energy generation by various national governments.

The keyword *activated sludge* came on top in 2016. The effect of pre-treatment methods on the microbial populations studied for converting waste-activated sludge to biogas (Xu et al. 2021). Methane-rich biogas production could be realised for both energy recovery from waste-activated sludge and pollution control.

*Biochar* was found to be a popular topic in 2021. Biochar as a cost-effective material has been applied to the improvement of biomethane production in anaerobic digesters for wastewater treatment. Biochar can be converted from biosolids. It is a carbon-rich form of charcoal that contains heavy metals and is an ideal catalyst for generating hydrogen from biogas (Patel et al. 2020). Research shows that biochar can significantly enhance the methane production rate (Wang et al. 2020). Biochar can also be applied as filters to replace mineral filters for the removal of pharmaceutically active compounds from wastewater (Jakub et al. 2019). It is particularly effective for onsite household wastewater treatment to treat wastewater that also contains pharmaceuticals and heavy metals. The use of biochar fits the circular economy and contributes to carbon emission reductions.

Much aligned with a global trend for research and policy in recent years, *circular economy* has also been gaining great momentum as a hot topic, especially in Europe, for wastewater to energy research (Marcal et al. 2021). The wastewater sector is transforming towards energy sustainability (Ghimire et al. 2021) and a circular economy (Guerra-Rodríguez et al. 2020) Recovery of energy is an important component of the circular economy, such as the biomethane produced from biogas (Bianco 2018). There are many WWTPs that have achieved energy self-sufficiency and generated up to 150% of the energy requirements (Guerra-Rodríguez et al. 2020).

The keyword analysis reveals how the global research focus of wastewater to energy changed over time and the recent progress of wastewater to energy, from wastewater treatment–centric to energy-centric and from technology-focused to more process/system-oriented. The research on wastewater to energy was extremely low in the early 2000s, indicated by the appearance of relevant keywords in publications less than five times per year until 2005. This research interest started to grow with a gradual expansion since 2009 and led to a significant spike in 2016. It appears that during 2009–2016, the research focus was largely concentrated on anaerobic wastewater treatment for biogas generation from both municipal and industrial (e.g. dairy and food production) wastewater treatment sources, and then the emphasis was shifted more to anaerobic co-digestion for biogas and methane production from 2017 onward. These also correlated with the trend of research on hydrogen production, which has become increasingly pronounced in ‘wastewater to energy’ research. Meanwhile, methane generation from waste-activated sludge and microbial communities appeared to be the popular research topics in 2019. Biogas upgrading for energy recovery was the research focus in 2020. The research focus shifted to target biochar and waste management with circular economy models and impact assessments in wastewater areas over the last 3 years. Biogas generation from wastewater as energy recovery is the key to adopting circular economy principles in the wastewater sector. Machine learning has been a rising research keyword in 2023. Research using machine learning has been focused on prediction and increased biogas production.

### Main research themes

**Fig. 9:**
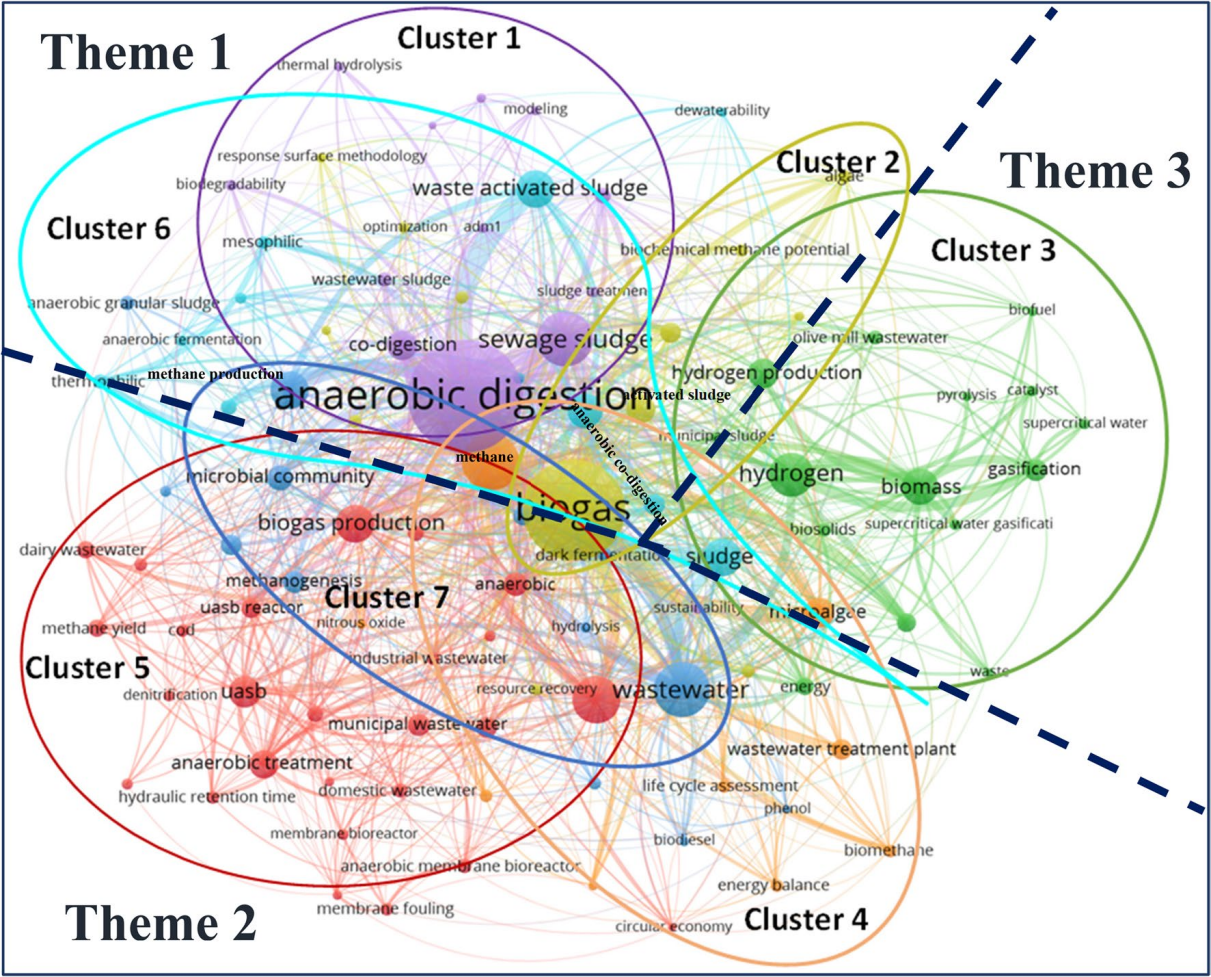
Co-occurrence network of keywords and clusters

In order to further identify and analyse the main research themes, a total of 100 author keywords from 2000 to 2023, selected based on a minimum of ten occurrences, were examined to create a co-occurrence network by using VOSviewer (Fig. 9). In this network, each node represents a keyword captured and the size of the node corresponds to the number of occurrences of that keyword. *Anaerobic digestion* appeared as the most used keyword, followed by *biogas*. Meanwhile, the arc that connects a pair of nodes denotes their relationship of co-occurrence, i.e. the thicker the arc, the more frequently the two keywords co-occur in the same context and thus the stronger their co-relation. For example, the occurrences of *anaerobic digestion* exhibited a strong association with *waste activated sludge* and *methane production*. Clusters can be defined and formed accordingly, illustrated in different colours, in relation to the strength of co-occurrences of those keywords. Overall, there are seven clusters identified within the co-occurrence network, with some overlapping with each other as shown in Fig. 9. The themes of research were then further derived for these clusters. Figure 9 presents an overview of the research trends of each cluster based on five main (most popular) keywords and the frequencies of their appearances in the publications from 2000 to 2023.

Cluster 1 (highlighted in purple) is centred on *Anaerobic digestion* with the inclusion of *sewage sludge* and *co-digestion* as well as some less-frequent keywords such as *biodegradability*, *bioenergy*, and *thermal hydrolysis*. It demonstrates the importance of research on bioenergy production from anaerobic co-digestion of sewage sludge. *Anaerobic digestion* as the most popular keyword in the cluster, together with *sewage sludge* and *co-digestion*, created the major research hotspot, which has seen a strong surge of interest since 2012. Sludge from municipal wastewater has been increasingly recognised as a renewable and cost-effective energy source for biofuels (Seiple et al. 2020). As a result, studies on *sewage sludge* have also been on an upward trajectory over the past 20 years.

Cluster 2 (in yellow) includes *biogas*, *activated sludge*, *pretreatment*, and keywords with fewer co-occurrences, such as *algae* and *energy recovery*. Gravitated towards *biogas*, it shows that there was intensive research on the pretreatment of activated sludge for biogas production. Pretreatment methods such as thermal pretreatment for anaerobic digestion cause the hydrolysis of sludge and provide substrate for the methanogen’s growth, thus increasing methane production (Ennouri et al. 2016). The most prominent topic in this cluster is *biogas*, which appeared far more frequently than other co-occurring keywords within the same cluster.

Cluster 3 (depicted in green) contains *hydrogen*, *hydrogen production*, *biomass*, *gasification*, and *energy* as the main keywords and largely overlaps with Cluster 2 around hydrogen production. Despite displaying no single dominant topic, these still pronounce the featuring research concentration of the cluster as hydrogen production from biomass gasification. Hydrogen production via biomass gasification was intensively studied (Salam et al. 2018) and reviewed (Cao et al. 2020). Biomass gasification is an indirect combustion technology that converts solid and liquid biomass into syngas including hydrogen.

Marked in light brown, Cluster 4 centres on the keyword *methane* and encompasses *microalgae*, *wastewater treatment plant*, *biomethane*, and *life cycle assessment*. The extent of correlations demonstrated by the network of these keywords signifies the emphasis of research on microalgal wastewater treatment for biogas production. Wastewater provides nutrients for microalgae growth, and the generated biomass can be used to produce methane (Caporgno et al. 2015).

Cluster 5 (in red) consists of the most frequent keywords of *wastewater treatment*, *biogas production*, *UASB* (upflow anaerobic sludge blanket reactor), and *anaerobic treatment*, as well as the less-frequent keyword—*energy recovery*. It suggests a coherent concentration of research on biogas production from wastewater treatment with the UASB. UASB is a form of an anaerobic digester that is used particularly to treat industrial wastewater and produce biogas with a high concentration of methane (Gür and Demirer 2019). The UASB system has a high rate of biogas production, needs low maintenance, and yields a low amount of sludge which can make it energy-self-sufficient (Ahmad and Senaidi 2023).

Cluster 6 (in light blue) contains keywords of *waste activated sludge*, *sludge*, *anaerobic co-digestion*, and low frequent keywords of *dewaterability*, *anaerobic fermentation*, and *food waste*. The connections among these keywords in the network indicate that the concentration of research was also on the generation of biogas from anaerobic co-digestion of waste-activated sludge. It is also noticed that a large proportion of Cluster 6 overlaps with Cluster 1, with shared accentuation on *anaerobic digestion* and *sludge*. Anaerobic digestion is versatile in treating waste-activated sludge in different conditions for biogas production in the anaerobic co-digestion, and simultaneous digestion processes were reviewed (Yang et al. 2019). Recent research shows anaerobic co-digestion of waste-activated sludge with other waste such as food waste at WWTPs is a promising approach for biogas production (Hallaji et al. 2019).

Cluster 7 (shaded in blue colour) involves high-occurrence keywords of *wastewater*, *methane production*, *microbial community*, and low-occurrence keywords of *granular sludge*, *methanogenesis*, and *biodegradation*. This indicates the intensive research on methane production from microbial communities in the wastewater. Methanogenesis of microbial communities plays a crucial role in anaerobic wastewater treatment, particularly for industrial wastewater. The relationships between industrial wastewater composition and methanogen microbial communities in anaerobic reactors were summarised by Vítězová et al. (2020).

The seven clusters were grouped into three themes based on the commonalities shared in the areas of research focus. Centred on ‘biogas generation from anaerobic digestion’ (Theme 1), Clusters 1, 2, and 6 belong to the same theme, with *anaerobic digestion* and *biogas* as the most dominant thematic keywords representing the research hotspots. Intensive research has been conducted on this theme, including anaerobic co-digestion of waste-activated sludge, for harvesting bioenergy and improvement of pre-treatment technologies used for activated sludge to produce biogas. Clusters 4, 5, and 7 have a common thematical emphasis on ‘methane production from microbial wastewater treatment’ (Theme 2). This theme encompasses studies on methane production using microalgae from wastewater treatment plants, energy recovery as the form of methane using various techniques such as UASB, and methanogenesis of the microbial community in the methane production of wastewater. The number of research publications in the scope of Theme 2 was relatively low in comparison to those related to Theme 1. However, the trend of research interest in biogas exhibits a very similar pattern to and coincides with that of the research publications on anaerobic digestion, making Theme 1 and Theme 2 the two primary themes of ‘wastewater to energy’ research. Cluster 3 represents a special theme, signifying ‘hydrogen production from biomass’ (Theme 3). Biomass gasification is a key focus of research on this theme. It is a relatively mature technology pathway to convert biomass to hydrogen without direct combustion to produce electricity or heat for wastewater treatment processes.

### Research hotspots

The analysis of occurrence patterns and cluster networks of author keywords in publications help to shed light on trends, themes, and hotspots for the wastewater-energy nexus, which help to answer the research questions of this study.

In terms of identifying the overall global trends over the past two decades, both the word-cloud analysis and the co-occurrence network of popular keywords lead to a convergence of findings that underscore the dominant focus in the ‘wastewater to energy’ research on biogas production through anaerobic digestion of sewage sludge. The extant research in this field was categorised into three typical themes based on the clusters of keyword network analysis, as shown in Fig. 9. The focuses of the three themes are consistent with the overall trend: biogas produced by the anaerobic digestion process of sewage sludge, methane production from microbial wastewater treatment, and hydrogen production from biomass. These research themes state the long-term goals of ‘wastewater to energy’ studies.

The analysis of the keywords revealed how the global research focus has evolved over the years, with the most research work produced since the year 2010. While ‘wastewater to energy’ gained attention in the early 2000s, the number of research publications remained extremely low. The research in this area has grown steadily since 2009 and then expanded rapidly around 2016. The research pre-2010 was limited and largely concentrated on sludge treatment to capture opportunities for energy recovery. Anaerobic digestion for biogas generation became the primary focus from 2010 and onward, which saw the first surge, both in volume and in significance, of “wastewater to energy” research, while methane and hydrogen production were also increasingly studied. There has been growing research interest in methane production from co-digestion since 2017. Pre-treatment and activated sludge have also emerged as hot topics since 2018. Based on the analysis of keyword occurrences and clusters, research hotspots attracting much progress in recent years, especially the last 3 years, were *activated sludge*, *biochar*, *biomethane*, *biogas upgrading*, and *circular economy*.

## Future directions of ‘wastewater to energy’ research

The analysis of global trends and patterns of research, indicated by the evolution of keywords and the thematic research focuses, helps to provide further insight to reveal and inform potential directions for future research in the following key areas:

- Optimisation of anaerobic digesters for biogas generation
- Biogas and biochar from anaerobic digestion systems
- Energy recovery for the circular economy of WWTPs
- New technologies for biomethane generation
- Hydrogen production from biogas, biosolids, and recycled wastewater
- Alignments with sustainable development goals, policies, and practices

### Optimisation of anaerobic digesters for biogas generation

The analysis of trends and patterns of research, indicated by the evolution of keywords, has *optimisation of anaerobic digesters* for biogas generation identified as a main area of development for ‘wastewater to energy’ research and applications. With significant advancement since 2010, anaerobic digestion has increasingly become a mature and widely accepted technology and can produce a high amount of electricity from wastewater (about 0.1 kW h/m^3^) (Stillwell et al. 2010). It is now commonly employed in large WWTPs (> 22,000 m^3^/day) because of the amount of available sludge for digestion and can meet on-site energy needs. Many European countries have financial incentives in place for the development and deployment of anaerobic digesters to produce heat and electricity from biogas (Maktabifard et al. 2018). Table 2 summarises some most recent and key studies on optimising anaerobic digestion technology and processes for generating biogas and energy products with respect to their research focus, application scale, advantages, and limitations.

**Table 2:** Summary of latest studies on biogas production from anaerobic digestion

Research focus	Process	Generated product	Study scale/type	Advantages	Limitations	Source
Production of hydrogen assists with biochar and activated char	Catalytic methane decomposition	Hydrogen	Lab/experimental scale	Increase methane conversion rate	Rapid blockages of active sites and surfaces of biochar and activated char can cause a rapid decline in methane conversion	(Patel et al. 2020)
Increasing anaerobic digestion efficiency using biochar	Anaerobic digestion	Methane	Experimental	Increase the production of digestion gas	Controlled conditions	(Shin et al. 2022)
Optimal technology selection for the biogas upgrading to biomethane	Biogas upgrading to biomethane	Methane	Desk-top modelling	The developed model can determine the optimal technology and operational conditions	Conceptual optimal design	(Martín-Hernández et al. 2020)
Thermal hydrolysis process as pre-treatment for electricity generation	Pre-treatment using thermal hydrolysis process for anaerobic digestion	Biogas	Pilot and demonstration scale	Increase the biogas conversion rate	Anaerobic digestion requires pre-defined feeding schedules	(Lafratta et al. 2020)
Predict anaerobic digestion performance using machine learning algorithms	Model development	Methane	Desk-top modelling	Accurate prediction of digester performance using machine learning algorithms	Small dataset of this study	(Freund et al. 2020)
Improve plant performance and biogas production	Optimisation of nitrogen removal	Biogas	Desk-top modelling/concept study	A range of scenarios are considered with potential	The concept of shifting aerobic to anaerobic sludge stabilisation needs to be tested for the specific case	(Vergara-Araya et al. 2022)
Estimation of biogas production	using data-driven approaches to optimise energy generation	Biogas	Desk-top modelling	The most accurate model was established	Models need to be developed using the specific conditions	(Asadi et al. 2020)
System optimisation for effective hydrogen production	Anaerobic digestion and biogas steam reforming	Hydrogen	Experimental	Increased biogas production	The reforming catalyst with resistance to sintering	(Park et al. 2020)
Energy recovery	Anaerobic digestion of algal biomass from integrated with microalgae/constructed wetland wastewater treatment	Methane	Pilot study	Promising electric energy generation potential	Need to test the proposed technology with different native species from diverse regions	(Silveira et al. 2023)
Estimation of the electrical energy produced by energy recovery	Model development	Biogas	Desk-top modelling	Electrical energy is predicted using only 3 parameters (gas flow, conductivity, and TSS)	Errors in the prediction need to be reduced	(Kerem and Yuce 2023)

### Biogas and biochar from anaerobic digestion systems

Improving *biogas recovery* from anaerobic digestion systems represents another hotspot for further research. Two main areas attracting increased research in this aspect are process enhancement through optimising key parameters such as digestion time, process stability, biogas yield and rate, and exploring the use of new and improved microbial inoculants (Zagorskis et al. 2023). Recent studies have been conducted on the optimisation of the systems for biogas generation from anaerobic digestion by increasing the organic content of feedstock (Freund et al. 2020), through modelling the energy generation with machine learning algorithms (Kerem and Yuce 2023), biogas steam reforming (Park et al. 2020), and combined optimisation with nitrogen removal (Vergara-Araya et al. 2022). These studies were mainly proof-of-concept studies of modelling using advanced computing such as machine learning to optimise the technology or predict the performance for effective biogas generation. In addition to biogas, *biochar* is also considered a product of wastewater treatment with strong commercial value for energy generation. Some studies in Table 2 emphasised converting biochar from biosolids and also having it applied as a catalyst to produce hydrogen from biogas on-site at WWTPs (Patel et al. 2020). Another focus for research is to produce biochar from anaerobic digestion and to further use it to couple with or modify the anaerobic digestion process for promoting methane production (Song et al. 2021), which signifies the potential of biochar’s functions to enhance the process of converting wastewater to energy. Biochar can be produced from sewage sludge and microalgae mixtures (Bolognesi et al. 2021) and biomass (Seow et al. 2021) to improve digestion efficiency by operating as a medium, contributing trace elements, and increasing the number of microorganisms (Qambrani et al. 2017).

### Energy recovery for circular economy practices of WWTPs

Energy recovery from wastewater is an essential part of the *circular economy* model (EEA 2022). Energy recovery can be achieved by anaerobic digestion of algal biomass from integrated microalgae/constructed wetland wastewater treatment (Silveira et al. 2023). The concept of circular economy can serve as the main impetus for WWTPs in the development of technologies towards energy and carbon neutrality (Zhang and Liu 2022) through resource recovery and carbon reduction (Preisner et al. 2022) allowing better management and utilisation of wastewater treatment processes towards energy self-sufficiency and resource efficiency. This not only helps waste reduction, but also turns waste streams into valuable inputs for other processes. Supported by the growing body of research in recent years, biogas produced from wastewater through anaerobic digestion becomes the main contributor to energy self-sufficiency at WWTPs (Llácer-Iglesias et al. 2021). While most large WWTPs have an anaerobic process on site, it is difficult for small-scale plants to achieve the same energy self-sufficiency. Technology such as solar-PV can provide an additional renewable energy boost to power the plants and aid the enhancement of cleaner energy production that contributes to the transition of WWTPs in achieving energy neutrality or even energy positive. Only a small number of WWTPs in the world are operating in the vicinity of being energy neutral, with the Sheboygan Regional Wastewater Treatment Facility in Wisconsin (USA) as one of the few small-scale WWTPs able to achieve nearly 100% energy self-sufficiency. Future research to identify the technology barrier to address the large gap between current practices and energy self-sufficiency needs for WWTPs is necessary, especially the generation of biogas with the input of renewable energy via solar (Baş and Köksal 2022) or wind (Gu et al. 2017) at small-scale WWTPs as well as increasing biogas production by adding other organic material, such as biosolids from other WWTPs or food waste (Kunatsa and Xia 2022). It is worth noting that future research on the utilisation of biochar as an aide for increasing anaerobic digestion will have added benefits for the circular economy (WSAA 2023), including the use of food-waste-based biochar to increase methane production (Kalengyo et al. 2023). This could be further expanded to focus on exploring the use of alternative feedstocks such as food waste (Paranjpe et al. 2023) or agriculture waste (Tshemese et al. 2023) to improve the efficiency of anaerobic digestion.

### New technologies for biomethane generation

Exploring and improving new technologies for *biomethane generation* from wastewater is trending as another recent key focal research area with substantial progresses in recent years, as epitomised in Table 3.

**Table 3:** Technologies for biomethane generation from wastewater

Technologies	Process	Typical Systems	Scale of the technology	Advantages	Limitations	Source
Anaerobic digestion	Biological process that converts organic matter in wastewater into biogas	anaerobic sequencing batch reactor, upflow anaerobic sludge blanket	Full scale	Can treat domestic sewage, industrial wastewater, and agricultural waste Can reduce the volume and mass of the organic waste Can reduce greenhouse gas emissions by capturing and utilising methane	Requires careful control of process conditions to maintain efficient operation, can generate odour, can produce a high concentration of residual sludge, and requires a long retention time	(Kumar et al. 2021)
Membrane bioreactors	Combined with anaerobic digestion	Consists of a bioreactor tank, a membrane filtration system, and a control system	Lab-scale and full-scale	High biogas production, reduced footprint, lower sludge production, lower odour emissions	High energy consumption, high capital costs, membrane fouling, sensitivity to shock loads	(Elmoutez et al. 2022)
Microbial Electrolysis Cells	microbes to catalyse the reduction of carbon dioxide (CO_2_) and other organic compounds to produce methane (CH_4_) through an electrochemical process	consists of an anode and a cathode separated by a membrane	Primarily lab-scale, some pilot-scale	High energy efficiency, reduced carbon footprint, flexibility	High cost, low methane yield, technical challenges	(Koul et al. 2022)
Upcycling (co-digestion)	Use other waste materials such as food waste or agricultural waste or algal biomass as feedstock	Involves collecting organic waste materials, and processing them through anaerobic digestion or microalgae cultivation on wastewater	From small to full	Waste reduction, local production, economic benefits	Technical challenges and high capital cost	(Tsapekos et al. 2021); (Vaz et al. 2023); (Deng et al. 2023)
Biogas Upgrading	Upgrade biogas to biomethane	Water scrubbing, pressure swing adsorption, membrane separation	Varying levels of maturity, with some being widely implemented and others still in developmental stages	Suitable for grid injection, flexible in handling various input gas compositions	Involve high capital and operational costs Chemical scrubbing can be energy-intensive	(Martín-Hernández et al. 2020); (Assunção et al. 2021)
Thermal hydrolysis	Pre-treatment process	Break down cell walls and complex organic molecules to make it easier for microbes to digest		Increase biogas production and reduce pathogen levels	Energy intensive and requires high capital investment	(Balasundaram et al. 2023)

Upgrading the lower-valued biogas to a higher-valued biomethane is a critical step of improvement that can result in greater circular economy benefits to support the wastewater treatment sector. Biomethane is converted by removing carbon dioxide through a biogas-upgrading process (Martín-Hernández et al. 2020) to produce methane with a purity level of 98% which is a higher-valued product as compared with the normal biogas (60% methane, 40% carbon dioxide, and other contaminants). Many WWTPs have utilised the anaerobic co-digestion process to generate surplus biogas and upgrade biogas to biomethane for the domestic market using various techniques including scrubbing (Martín-Hernández, 2020), adsorption, membrane separation, and cryogenic technology (Assunção et al. 2021). Water scrubbing is the most applied approach for biogas upgrading because of low capital and operating costs. Nonetheless, with the rapid market growth and progress of membrane separation technology, it is expected to be the dominant technology in the near future (Nguyen et al. 2021). Further research is needed on emerging technology for biogas upgrading, optimising, and exploring the application of the membrane separation technique would be an achievable option without too much further development. It is also essential for future research to focus on process development and optimisation to lower both capital and energy costs. Combining anaerobic digestion and membrane bioreactor appears the most compelling technological option in the near future. Other technologies, such as microbial electrolysis cell technology, are also worth further investments to move from laboratory-scale trails to full-scale applications.

### Hydrogen production from biogas, biosolids, and recovered wastewater

The patterns of recent progress in ‘wastewater to energy’ research have also featured a heightening interest in *hydrogen* and *hydrogen production*. This signifies one of the most important future research areas from the trends analysis and the critical decarbonising role of hydrogen in the wastewater industry. Growing studies on technologies for hydrogen production using biogas or biosolids as by-products of wastewater treatments have been observed since the 2000s. Much of the research, however, was limited to a lab- or pilot-level. While most of the technologies for hydrogen production from wastewater are yet to be attested as economically viable for large-scale deployments, this is an active area of research with some promising recent progress (Batool and Shahzad 2021). There are a series of low-carbon footprint technologies for hydrogen production from wastewater including fermentation (Zhang et al. 2022) and photocatalysis. Yet there are significant challenges towards commercial use such as the design and configuration of photoreactor for optimal light absorption (Elgarahy et al. 2022), and catalyst fouling is also a difficulty (Islam et al. 2021). Future research is needed to close the gap between demonstration as proof of concept and actual industry-based applications. More research is needed to develop production from wastewater (Arun et al. 2024). Future research could focus on scaling up these processes and exploring commercialisation opportunities.

Globally, research on the nexus between wastewater and hydrogen production is further supported by strategies and planned investments of governments and industries in many countries for achieving the net zero targets (Meadowcroft and Rosenbloom 2023). One approach for hydrogen production from wastewater is through electrolysis of recycled water using renewable energy to reduce carbon emissions. It consumes the treated wastewater as non-potable water to generate hydrogen and a pure oxygen co-product. The co-product can be recycled to enhance aerobic wastewater treatment on site. This is the preferred approach for the wastewater industry as electrolysis is a relatively mature technology and it is well fit in with the circular economy by cutting carbon emissions (Freund et al. 2020) and recovery energy through hydrogen production (Simoes et al. 2021). However, there are still knowledge gaps on how the impurities in recycled water affect water electrolyser design and process operation. Future research needs to assess the suitability of recycled water for water electrolysis (Woods et al. 2022) and evaluate the influence of recycled water on the performance of water electrolysers and guidelines for the design of integrated water electrolysers and existing wastewater treatment plants for hydrogen production. The other favourite method of hydrogen production is steaming biogas reformation to separate pure hydrogen from the syngas generated by the direct conversion of methane in raw biogas from the anaerobic digestion of sludge. This method has significant potential but is in the early development phase and needs intensive research for hydrogen production from biogas. Future studies are needed on the enhancement of hydrogen production from biogas sourced from wastewater treatment plants. Microbial electrolysis cells can efficiently convert organic waste in wastewater into hydrogen with a lower energy input compared to traditional methods, while simultaneously treating the wastewater (Arun et al. 2024). Microbial electrolysis cells are currently limited by their catalysts, reactor design, anode and cathode materials, and challenges in scaling up for industrial use (Aber et al. 2023).

### Alignments with sustainable development goals, policies, and practices

‘Wastewater to energy’ research initiatives are well aligned with multiple Sustainable Development Goals (SDGs) set by the United Nations (Kalengyo et al. 2023). Key SDGs contributed by ‘wastewater to energy’ include SDG 6 (Clean Water and Sanitation) by reducing pollution and increasing water recycling and safe reuse, SDG 7 (Affordable and Clean Energy) through the generation of renewable energy from wastewater, contributing to more sustainable and affordable energy sources, and SDG 13 (Climate Action) by reducing greenhouse gas emissions from wastewater treatment processes and utilising energy recovery methods. Reduction of energy consumption in WWTPs is of critical importance to sustaining urban wastewater treatment and reuse (Silva 2023).

Improving energy efficiency holds immense potential for transformation towards greenhouse gas emissions reduction and climate change mitigation in achieving the SDGs. Decarbonisation of wastewater treatment involves minimising the carbon footprint of treatment processes (Rusmanis et al. 2022). This can be achieved through renewable energy integration, optimization of treatment processes, and adoption of low-carbon technologies. Based on the discussion above, we propose three key strategies to achieve net-zero carbon along with energy sufficiency in the water sector and to contribute to SDGs. First, on-site integration of renewables such as solar and wind energy to maximise on-site renewable capacities and biogas upgrading. Biogas can be used for combined heat and power to decrease energy consumption and carbon emissions. It could support operations across the wastewater treatment facility to provide power to the grid. Flexibility in adopting technologies with process automation and integration with existing technologies to increase overall efficiency. Anaerobic digestion equipped with combined heat and power facilities and installation of energy-extracting technologies will contribute to the energy self-sufficiency of WWTPs. The on-site installation of standalone or hybrid renewable energy generators with energy storage could provide a decarbonised power source for wastewater treatment systems. secondly, research and innovation in wastewater-to-energy technologies for energy security and self-sufficiency through closed-loop inputs and outputs should be supported by policies, including pilot projects, demonstration programs, and collaboration with the private sector. Encouraging innovation can lead to more cost-effective and environmentally friendly solutions. One example of research innovation is fuel cells which have gained popularity for generating electricity (Rani et al. 2022). The application of modern treatment techniques like microbial fuel cells (Kunwar et al. 2023) and microbial electrolysis cells (Deng et al. 2023) can enable the conversion of wastewater’s chemical energy into electricity without external energy input, leading to significant energy savings and potential energy recovery. The last one is the modelling of the effects of different scenarios for energy optimisation and generation with data analytics using artificial intelligence and machine learning. Such studies could be using models to calculate and adjust parameters for the biogas production from wastewater such as anaerobic digestion (Chaabna and Semcheddine 2023) since it is easier and cheaper to test a control method on simulation than to test it on a real system. Studies could also use a hydroeconomic model to assess the tradeoffs between different wastewater management approaches (Kuwayama and Olmstead 2020).

## Conclusion

This paper presents a structured bibliometric analysis and review on the research publications recorded in the Web of Science database from 2000 to 2023 to methodically examine the development of the ‘wastewater to energy’ research field and to identify global trends, potential hotspots, and future research directions. The study highlights three main research themes in ‘wastewater to energy’, which are biogas production through anaerobic digestion of sewage sludge, methane generation from microbial wastewater treatment, and hydrogen production from biomass. The findings also indicate that activated sludge, biochar, biomethane, biogas upgrading, and circular economy represent key topics increasingly gaining momentum in recent research publications. These trends align with global research patterns, highlighting the exploration of biochar applications, economic and environmental assessments of biochar production, membrane techniques for biogas upgrading, small-scale biogas generation, and renewable energy integration for both operational performance and energy self-sufficiency of wastewater treatment plants. The analysis also signifies transforming the traditionally energy-consuming and carbon-intensive water treatment process into a significant producer of energy, recycled water, and biosolids, contributing to SDGs and circular economy practices through lowering greenhouse gas emissions and adopting energy recovery methods. The review of recent key research publications further reveals the importance of resource recovery, including biosolids and biochar, for reducing waste and converting it into valuable inputs. In light of policy and practice implications, three strategies are envisaged as critical for achieving the net-zero target in the wastewater sector, i.e. integration of on-site renewables and biogas upgrading for energy self-sufficiency, optimising energy recovery from wastewater treatment systems, and fostering research and innovation in ‘wastewater to energy’ supported by policy incentives. These strategies encompass advanced technologies, such as microbial fuel cells, electrolysis cells, and anaerobic digestion for biogas production, and provide future research thrusts to enhance the uptake of data analytics, artificial intelligence, and machine learning for a holistic approach towards sustainable and efficient wastewater treatment.

Nevertheless, the capacity and the results of the analysis are subjected to limitations in both data sources and techniques applied. As the sampling of data for this study was predominantly from the Web of Science archives, the investigation could have been affected by a potential bias from a single database. Future analysis can incorporate other research databases and data achieves to improve the scope of analysis and the representativeness of findings. Also, interrogating article citation metrics together with the keyword analysis is able to enhance the forecasting of future research trends. Applying machine learning techniques can help further improve the rigour and the quality of such analysis.

## Data Availability

The data and materials that support the findings of this study are available on request.
